# Contemporary evaluation of women and girls with abnormal uterine bleeding: FIGO Systems 1 and 2

**DOI:** 10.1002/ijgo.14946

**Published:** 2023-08-04

**Authors:** Varsha Jain, Malcolm G. Munro, Hilary O. D. Critchley

**Affiliations:** ^1^ Centre for Reproductive Health University of Edinburgh Edinburgh, Scotland UK; ^2^ Department of Obstetrics and Gynecology David Geffen School of Medicine at UCLA Los Angeles California USA

**Keywords:** abnormal uterine bleeding, heavy menstrual bleeding, iron deficiency, iron deficiency anemia

## Abstract

Abnormal uterine bleeding (AUB) is common, often debilitating, and may affect over 50% of reproductive‐aged women and girls. Whereas AUB is a collection of symptoms that include intermenstrual bleeding and abnormalities in period duration, cycle length, and regularity, it is heavy menstrual bleeding (HMB) that is most contributory to iron deficiency and related anemia. It is apparent that AUB, in general, and HMB, in particular, remain underrecognized and underreported. FIGO created two systems for assessing and classifying AUB. FIGO System 1 defines the bleeding pattern using four primary descriptors: frequency, duration, regularity, and flow volume. FIGO System 2 provides a structured classification system of possible causes of AUB, using the acronym PALM‐COEIN. “PALM” refers to structural causes of AUB (Polyp, Adenomyosis, Leiomyoma, Malignancy), and “COEI” refers to nonstructural causes (Coagulopathy, Ovulatory dysfunction, Endometrial, and Iatrogenic). The “N” is reserved for those entities that are currently not otherwise classified. Using FIGO System 1 as a gateway to FIGO System 2 streamlines the investigation of reproductive‐aged women and girls with AUB. Understanding the pathogenesis of the FIGO System 2 “PALM‐COEIN” causes helps interpret investigations and the onward management of AUB. Numerous evidence gaps exist concerning AUB; however, if researchers and trialists universally adopt FIGO Systems 1 and 2 for the assessment and diagnosis of AUB, clear translatable research findings can be applied globally.

## Synopsis

Abnormal uterine bleeding and its associated conditions (iron deficiency with/without anemia) are underrecognized and underreported, requiring precise, systematic assessment using FIGO systems 1 and 2.

## 1 INTRODUCTION

### 1.1 Menstruation and abnormal uterine bleeding

Nongestational abnormal uterine bleeding (AUB) in the reproductive years usually originates in the endometrium, regardless of the primary cause. Abnormal uterine bleeding is an umbrella term^1^ that includes heavy (HMB), irregular, and intermenstrual bleeding (IMB), as well as disorders of cycle length—those more or less frequent than the normal range of 24–38 days. Understanding the physiology of normal endometrial function aids understanding of why the causes of AUB lead to altered endometrial behavior (i.e. one of altered bleeding characteristics) and provides a guide to interpreting investigational findings in a fashion that facilitates the creation of a personalized menu of therapeutic options.

Almost half of the world's human population will menstruate^2^ and during their reproductive years, it has been estimated that at least one in three women will experience AUB.^3^ However, recent questionnaire‐based surveys have found that HMB alone may be prevalent in over 50% of women of reproductive age.^4^, ^5^ This would suggest that the lifetime risk estimation of AUB affecting one in three women is likely to be a gross underestimation. For decades, it has been reported that women experience many barriers to receiving care for AUB.^6^ This may account for the discrepancies in “patient data”‐based studies that suggest the one in three lifetime risk of AUB, whereas “direct‐to‐patient” studies suggest the much higher point prevalence of over 50% for HMB alone.^7^

It has long been known that the symptoms that comprise AUB may have debilitating effects on reproductive‐aged women and girls.^8^ Even in antiquity, over 2400 years ago, Hippocrates acknowledged that HMB is associated with concomitant disease.^9^ In the 17th century, Thomas Sydenham described the weakness and pallor of anemia as a consequence of what we now call HMB.^10^ Despite these early observations, and in a world where approximately 800 million women are menstruating, the combined issues of HMB and associated iron deficiency (ID) and iron deficiency anemia (IDA) are understudied and underrepresented.^11^

The taboo surrounding menstruation stems from societal, religious, and cultural ideologies^12^ and may be based on false information and prejudice. These influences further hinder the progression of research and the dissemination of information, which is much needed concerning menstruation. Therefore, it is vital to acknowledge the importance of accurately understanding how to evaluate women and girls with AUB holistically, considering its impact on quality of life and any concomitant ID or IDA that can occur secondary to HMB. With this approach, it may be easier to address the taboos many women face, normalize the conversation around menstruation, and improve the care women receive for their AUB. This paper focuses on the evaluation of AUB and its possible causes.

## 2 THE ENDOMETRIUM

### 2.1 Role in nongestational AUB in the reproductive years

The endometrium is a multicellular dynamic tissue that resides within the uterine corpus and lines the endometrial cavity. Its functions in the reproductive years include reception and implantation of the embryo, ongoing support and maintenance of pregnancy, and, in the absence of a pregnancy, to shed (i.e. to menstruate) and subsequently undergo repair.^3^ Multiple processes are involved to ensure that efficient endometrial shedding and repair occur cyclically, with coordinated actions that prevent scarring. Progesterone withdrawal refers to the fall in circulating progesterone concentrations due to the demise of the corpus luteum in the absence of pregnancy and is the trigger for menstruation, setting in motion an organized cascade of local events that control the cessation of endometrial bleeding.^13^, ^14^ To prevent future loss of function and appropriate menstrual blood loss, the endometrium undergoes several carefully coordinated processes including, but not limited to, vasoconstriction, maintenance of physiologic clots, and endometrial repair that ensure timely endometrial hemostasis.^15^ Vasoconstriction within the endometrial vasculature, particularly the spiral arterioles, directly reduces menstrual blood loss. Key regulators for effective vasoconstriction in the endometrium include prostaglandin F2α (PGF2α) and endothelin‐1 (ET‐1).^16^, ^17^ In addition to the physical reduction of blood loss, vasoconstriction induces a hypoxic microenvironment within the upper functional zone of the endometrium that is shed at menses. This hypoxia stabilizes the alpha subunit of hypoxia‐inducible factor (HIF), which in turn leads to functional adaptations within the endometrium to control blood loss, including the generation of endometrial repair factors, such as vascular endothelial growth factor (VEGF), adrenomedullin, and connective tissue growth factor (CTGF).^18^ Alongside these processes, the initiation of the clotting cascade leads to the development of fibrin clots within endometrial blood vessels, which are also crucial in controlling menstrual blood loss. Degradation of the fibrin clots via the fibrinolytic system occurs promptly to ensure ongoing functionality of the endometrium. Components of the fibrinolytic system, including plasminogen activator inhibitor‐1 (PAI‐1), thrombomodulin (TM), and antithrombin III (ATIII) have all been found within human endometrium.^19^, ^20^

In the endometrium of a woman with HMB, there is likely to be dysregulation in the above‐mentioned homeostatic control mechanisms, whereby coordinated events no longer occur in a synchronized fashion, and menstrual blood loss therefore increases. An example is the events that occur in the endometrium of a woman with leiomyomas (AUB‐L, uterine fibroids). Endometrium investigated from women with leiomyomas has shown paradoxically higher levels of PGF2α, yet decreased levels of endothelin receptors,^21^, ^22^ potentially leading to faulty vasoconstriction and HMB. Transforming growth factor beta 3 (TGFβ3) is produced in excess quantities in the endometrium from women with submucous leiomyomas, reducing endometrial levels of PAI‐1, TM, and ATIII. This shift in plasminogen modulators may contribute to disturbed endometrial hemostasis and, thus, HMB.^23^ These changes may suggest a secondary disorder within the endometrium due to the presence of leiomyomas within the uterus, as opposed to a primary disorder within the endometrium (AUB‐E), which would occur without the presence of any other cause of AUB.^12^

Unfortunately, the currently available evidence regarding endometrium from women with leiomyomas does not adequately identify the impact the location of the leiomyoma (submucous versus other) has on the altered behavior of the endometrium. While there exists evidence that submucous leiomyomas are associated with global endometrial changes in molecular expressions associated with endometrial receptivity, not seen in women with intramural leiomyomas or no leiomyomas at all,^24^ similar comparative data do not exist for the molecular expressions associated with the mechanisms of local endometrial hemostasis. While we suspect that the adverse impact of leiomyomas on endometrial hemostasis will be greater or even limited to those leiomyomas lying adjacent to the endometrium, confirmatory evidence is still lacking. Studies of this nature have been conducted with the primary focus on endometrial receptivity, and therefore the ability to relate these findings to the endometrium from women with the complaint of AUB is also unknown. Consequently, this knowledge gap warrants further investigation to fully understand the pathophysiology of leiomyoma‐associated HMB (AUB‐L) in a fashion that better informs treatment decisions.

While women with leiomyomas may display local molecular and functional irregularities within the endometrium, women with a coagulation disorder (e.g. von Willebrand disease) have a systemic impairment that has an adverse impact on the hemostatic control mechanisms within the endometrium, therefore leading to HMB.^25^ It is possible for women to have multiple causes of AUB, thus potentially synergistically influencing the hemostatic control process within the endometrium, leading to HMB. Interestingly, many causes of AUB increase in prevalence with advancing age, yet the molecular and cellular changes that occur in the endometrium as a direct result of aging remain unknown.^26^, ^27^ Further research to elucidate the normal physiology of the endometrium through the reproductive life cycle is essential.

## 3 THE VALUE OF THE TWO FIGO SYSTEMS

### 3.1 Characterization and classification

Inconsistent terminology related to AUB was—and still is—pervasive in clinical care and the design and interpretation of basic, translational, clinical, and epidemiological research. This circumstance adversely affects the construct and interpretation of systematic reviews and meta‐analyses and, in turn, has a negative effect on clinical care, patient education and counseling, and the training of medical students, residents or registrars, and those in other postgraduate programs.

To systemize how women with AUB are evaluated, the vital step of improving and standardizing terminology used within the field was undertaken by FIGO after an extensive multistage process.^28^ International consensus using a Delphi process was sought from 2005 onward, leading to the creation of the two FIGO systems for the assessment and classification of AUB.^29^ By standardizing the descriptions of normal and abnormal menstrual function and the classification of causes of nongestational AUB in the reproductive years, communication regarding nongestational AUB can improve. Standardization fosters the creation of more homogenous populations with similar clinical features and functional and structural causes of AUB in a fashion that enhances the design and interpretation of research, facilitates education, and allows for more evidence‐based and consistent counseling of affected women regarding appropriate options.

Nongestational AUB in the reproductive years can be either acute or chronic. Chronic AUB is defined by FIGO as: “bleeding from the uterine corpus that is abnormal in volume, regularity, and/or timing, and has been present for the majority of the past 6 months.” In contrast, acute AUB is: “an episode of heavy bleeding that, in the opinion of the clinician, is of sufficient quantity to require immediate intervention to prevent further blood loss”.^30^

The structured approach to diagnosis embodied in FIGO AUB Systems 1 and 2 applies to both acute and chronic AUB; however, when acute, the clinician must act expeditiously to stem the bleeding, often before a complete evaluation is possible. With both acute and chronic AUB, it is also important to remember the concurrent effect on the body's iron stores, the risk of ID and IDA, and the potential and related impacts on the patient's overall health and quality of life. It is, therefore, essential to investigate iron stores and the possibility of IDA alongside the AUB evaluation and process as guided by FIGO Systems 1 and 2.

## 4 FIGO SYSTEM 1

### 4.1 Normal and abnormal uterine bleeding definitions and terminology

**FIGURE 1:**
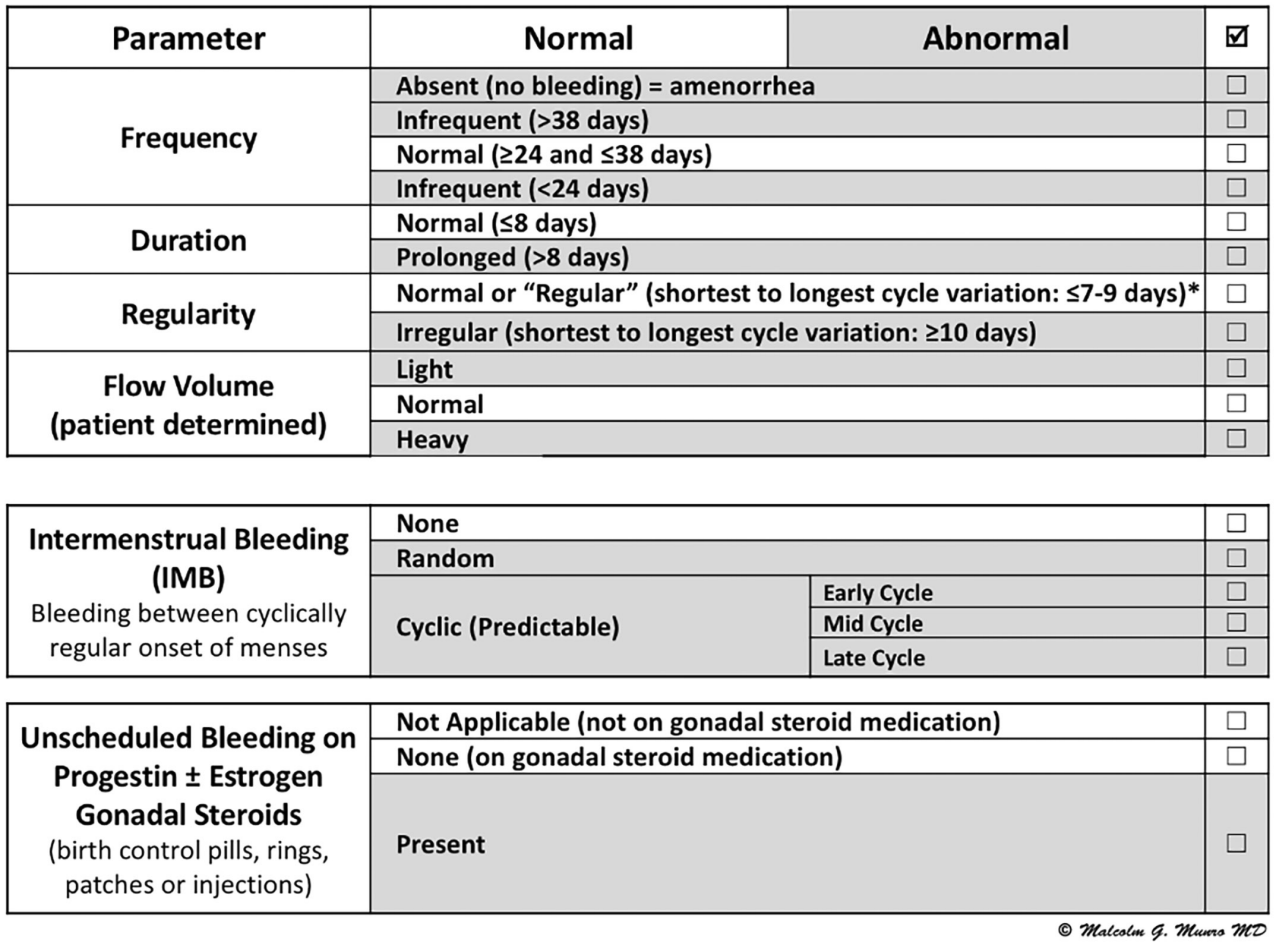
FIGO Abnormal Uterine Bleeding (AUB) System 1, defining the nomenclature and definition of AUB (30). *The normal range is age dependent, with shortest to longest days for 18–25 years at ≤9 days, 26–41 years at ≤7 days, and 42–45 years at ≤9 days.

FIGO System 1 was developed to formalize the nomenclature and definitions of normal and abnormal menstrual bleeding, allowing clinicians, investigators, and patients to align on the description of the bleeding patterns of those complaining of AUB (Figure 1). Each element is considered in the context of a structured history and defines normal and, therefore, abnormal features generally based on the fifth to 95th percentiles from large‐scale clinical studies. System 1 was the product of a rigorous Delphi process that was used to develop simple and translatable definitions while identifying and removing the need for obsolete terminologies such as menorrhagia, menometrorrhagia, and dysfunctional uterine bleeding.

As seen in Figure 1, application of FIGO System 1 in a clinical scenario requires a structured history that allows a description of the four parameters of frequency, duration, regularity, and subjective flow volume, as well as the presence or absence of IMB and unscheduled bleeding for those on progestin‐based gonadal steroid formulations. Of course, cyclical administration of estrogen and progestin‐containing agents, typically for contraception, is expected to result in cyclical withdrawal bleeding from the endometrium. All the metrics are based on the previous 6 months, provided there has been no pregnancy or puerperium.

#### Frequency

The number of days in an average menstrual cycle from the first day of bleeding in one cycle to the first day of the next cycle. The normal length of a menstrual cycle ranges from 24 to 38 days, whereas abnormal frequency of menstruation is less than 24 days (frequent menses) or more than 38 days (infrequent menses). The absence of menstrual bleeding, without an iatrogenic cause, should be characterized as amenorrhea, as this too is abnormal.

#### Duration

The number of consecutive bleeding days in each cycle, where the count begins with the onset of bleeding and ends on the last day of blood flow, including the light bleeding, often called spotting. It is normal for women to bleed for up to eight consecutive days, whereas longer than this would be considered prolonged and abnormal.

#### Regularity

The variance in days between the shortest and longest cycles defines regularity, which varies with age; below the age of 18 and after 45 years, ovulatory disorders are frequent and norms are not well defined. For women aged 18–25 years and those 42–45 years of age, cycle length can vary by up to 9 days. For those aged 26–41 years, the range is smaller—up to 7 days.^31^ Should the variance in cycle length fall outside these norms, the individual is said to have an irregular cycle, which typically reflects an ovulatory disorder.

#### Flow volume

In clinical medicine, and even for some investigational trials, flow volume is a subjective measurement and therefore is patient determined. As flow volume may change over the reproductive years, it is helpful to enquire about flow volume both historically and currently. In FIGO System 1, volume is categorized as normal, light, or heavy, per the patient's determination. FIGO adopted a definition of HMB that was first developed and published by the UK's National Institute for Health and Care Excellence (NICE) in 2007.^32^

#### Intermenstrual bleeding (IMB)

The presence of any bleeding between cyclically regular menses is considered abnormal. Intermenstrual bleeding can occur randomly or have a predictable nature depending on when in the cycle it occurs; early cycle (follicular phase before ovulation), midcycle (periovulatory), or late cycle (presumably in the luteal phase).

#### Unscheduled bleeding on agents that suppress or alter gonadal steroid production

Estrogen‐ and progestin‐containing contraceptive steroids may be administered continuously or cyclically; cyclical use is expected to result in predictable withdrawal bleeding. However, unscheduled bleeding while using these agents, such as birth control pills, rings, patches, or injections, is considered abnormal since they are designed to suppress or alter ovulatory and endometrial function. Other pharmaceutical agents in this category include gonadotropin‐releasing hormones and selective progesterone receptor modulators.

### 4.2 FIGO System 1 in practice

By structuring the menstrual history, FIGO System 1 serves as an essential gateway for the rest of the evaluation as it provides invaluable insight into the possible causes of AUB. For example, regular cycles with normal frequency suggest the presence of ovulation, while the irregular onset of menses suggests ovulatory dysfunction (AUB‐O). Intermenstrual bleeding may indicate the presence of a focal lesion like a polyp (AUB‐P), and the clinician should consider evaluation for malignancy or endometrial hyperplasia, as appropriate, given the clinical context (AUB‐M).^33^ If IMB is consistently early in the cycle (in the follicular phase) and typically comprises dark spotting or bleeding, a cesarean scar defect may be present and contribute to the symptom.^34^ The symptom of HMB in the context of apparently ovulatory cycles likely reflects a disorder of hemostasis, be it systemic (AUB‐C, further discussed in Section 5) or local, and if local, a primary endometrial disorder (AUB‐E) or one secondary to the presence of adenomyosis (AUB‐A) or a leiomyoma (AUB‐L, uterine fibroids), most commonly one that is submucous in location (AUB‐L_SM_). Regardless of the underlying cause, HMB is frequently associated with ID and IDA and related symptoms such as fatigue and reduced physical and cognitive function.

The structured history required for FIGO System 1 allows the clinician insight into disorders that may have been normalized by women and girls based on family, friends, or other healthcare practitioners. This may be especially true for HMB, which many women may have lived with unnecessarily, including the adverse impacts on quality of life.

## 5 FIGO SYSTEM 2

### 5.1 Classification (PALM‐COEIN)

FIGO's AUB System 2 was first published in the peer‐reviewed literature in 2011^30^ and later updated in 2018.^35^ It serves to categorize the potential causes or contributors to a patient's AUB symptoms and has been presented as a two‐part acronym that encompasses structural (“PALM”) and nonstructural (“COEI”) entities, as well as a category for potential contributors that are not otherwise classified (N).

**FIGURE 2:**
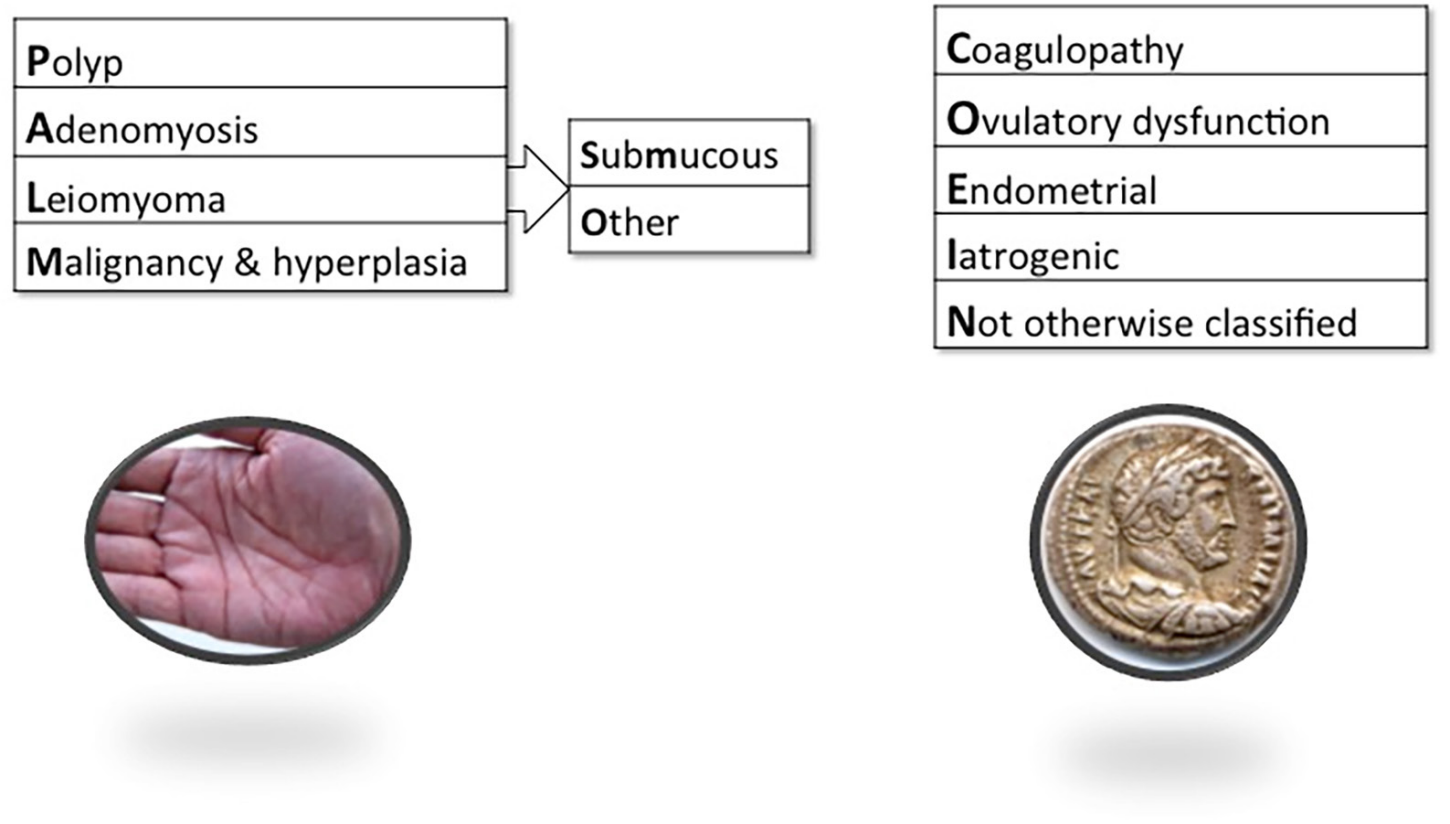
FIGO Abnormal Uterine Bleeding (AUB) System 2, describing the PALM‐COEIN Classification System for Causes of AUB in the Reproductive Years.^30^ Four categories define visually objective structural criteria (PALM: Polyp; Adenomyosis; Leiomyoma; and Malignancy and hyperplasia), four are nonstructural anomalies (COEI: Coagulopathy; Ovulatory dysfunction; Endometrial disorders; Iatrogenic causes), and one (N) is reserved for entities categorized as “Not otherwise classified.” The leiomyoma category (L) is subdivided into patients with at least one submucous myoma (LSM) and those with leiomyomas that do not impact the endometrial cavity (Lo).

The “PALM” group comprises polyps, adenomyosis, leiomyomas (uterine fibroids), and malignancy, including atypical endometrial hyperplasia or epithelial intraepithelial neoplasia. The “COEI” contributors include coagulopathy, ovulatory disorders, endometrial dysfunction (which may be considered primary and not related to the presence of any other causes of AUB), and iatrogenic causes (Figure 2). Modifications in 2018 moved anticoagulants from AUB‐C and agents causing ovulatory disorders from AUB‐O to AUB‐I.

It is essential to perform an appropriately thorough evaluation of women and girls with chronic AUB. Each potential cause or contributor may be found in isolation or may be present alongside other possible causes of AUB, and it is important to understand that many structural causes, including polyps, adenomyosis, and most leiomyomas, may indeed be asymptomatic and unrelated to the patient's symptoms. Consequently, even when a potential structural cause is identified, it is important to complete an assessment for nonstructural causes of AUB, which may be the main contributor to the patient's symptoms. One of the main tools to assist in this assessment is a structured history that complements the history taken for FIGO System 1. An example of a necessary aspect of the structured history includes a screening tool for coagulopathies (i.e. AUB‐C). FIGO recommends a screening tool that is 90% sensitive for the detection of a coagulation disorder. This includes an assessment to understand if HMB has been present since menarche and a combination of the following: previous postpartum hemorrhage, surgical or dental‐related bleeding, bruising (at least 1–2 times a month), epistaxis (nosebleeds; 1–2 times a month), frequent gum bleeding, and/or a family history of bleeding symptoms.^35^, ^36^ If positive, it warrants further investigation with blood tests such as von Willebrand factor, ristocetin cofactor, activated partial thromboplastin time (aPTT), or platelet function testing either with or without the support of a hematologist, dependent on the local availability of expertise. Other nonstructural causes of AUB require targeted questions within a structured history specific to the suspected underlying cause.

When more than one structural cause is identified, such as adenomyosis and leiomyomas, interpretation of investigations can be challenging when determining the options for management. Understanding the pathogenesis of the causes of AUB will assist with the interpretation of the investigation.

### 5.2 Relationship between pathogenesis and clinical investigations: Structural causes of AUB

#### Polyps (AUB‐P)

Uterine polyps may be present in the cervical canal but are most commonly “…focal endometrial outgrowths that can occur anywhere in the uterine cavity. They contain a variable amount of glands, stroma, and blood vessels…”.^37^ The size, texture, position, and number of polyps can all vary by the patient—features that may influence symptomology. Whereas the exact pathogenesis and natural history remain unclear,^38^ polyps are considered to arise from areas of epithelial and stromal overgrowth.^37^

The macroscopic diagnosis of polyps can be made via two‐dimensional (2D) transvaginal ultrasound (TVUS), where polyps appear as hypoechogenic lesions and cystic glands may be present. Diagnostic accuracy can be increased with color Doppler, three‐dimensional (3D) technology, and especially contrast—a process called sonohysterography—where saline or gel media instilled into the endometrial cavity provides sonographic contrast.^39^ Whether with 2D or 3D sonography, this approach is very accurate in diagnosing intracavity uterine lesions such as polyps or leiomyomas (sensitivity 96.9% and 99.5%, respectively).^40^ Importantly, meta‐analysis suggests that there is no significant difference in accuracy in the identification of intracavity lesions compared with hysteroscopy as a reference standard [40]. For premenopausal women with symptomatic polyps, the risk of malignancy is approximately 1%^41^; however, the number of participants used to delineate this value was small. AUB with polyps is seen to be a risk factor for malignancy, and therefore this warrants further investigation.^33^, ^41^, ^42^, ^43^

#### Adenomyosis (AUB‐A)

Adenomyosis is defined as the presence of endometrium‐like glands and stroma within the myometrium, typically surrounded by smooth muscle hyperplasia and/or hypertrophy. Recent evidence suggests that markers of fibrosis can be found in adenomyotic lesions.^44^, ^45^ The exact pathogenic mechanisms remain unclear, but include congenital or acquired extension into the myometrium from the endometrium, invasion from external endometriosis, and derivation from either embryonic or adult stem cells.^46^ It is unknown exactly how adenomyosis impacts the hemostatic control mechanisms within the endometrium to lead to abnormal bleeding; however, changes have been detected in the uteri of women with adenomyosis, which may suggest mechanisms that lead to HMB. Adenomyosis is a sex steroid‐dependent condition, and the local hyperestrogenism within adenomyotic lesions may influence the microenvironment and exacerbate the peristaltic movement of the subendometrial myometrium.^47^ This could activate the tissue injury and repair (TIAR) mechanism, thereby further increasing the local production of estrogen.^48^ There is increased estrogen receptor and decreased progesterone receptor expression in the eutopic endometrium in patients with adenomyosis, with potential contribution to progesterone resistance.^49^, ^50^ As the menstrual cycle is carefully controlled via a balance of these sex steroid hormones, these imbalances created through adenomyosis could impact the normal functioning of the endometrium. Further to this, there is evidence of enhanced angiogenesis both in adenomyotic lesions and eutopic endometrium via a combination of increased activin A, increased HIF‐1α levels, and increased VEGF levels and a reduction in antiangiogenic factors; for example, retinoid‐interferon‐induced mortality 19 (GRIM19).^51^, ^52^, ^53^, ^54^ Evidence suggests an activation of the coagulation and fibrinolysis systems within adenomyotic lesions, which may also increase the menstrual blood lost by women with adenomyosis.^55^ Finally, endothelial nitric oxide synthase (eNOS) and tissue factor are elevated in the eutopic endometrium from women with adenomyosis, and these changes are associated with HMB.^56^, ^57^ A better understanding of the pathogenesis of this enigmatic condition could allow for a classification of adenomyosis and more targeted treatment.

While histopathologic analysis of the myometrium has been the gold standard for the diagnosis of adenomyosis, usually following hysterectomy, such an approach is not applicable to women wishing to preserve their uterus. In the past 20 years, noninvasive methods have been shown to be relatively sensitive and specific for the diagnosis.

Two‐dimensional, 3D, and color Doppler TVUS can be used to detect adenomyosis with a sensitivity of approximately 84%, 85%, and 94%, respectively.^58^, ^59^ There are several features suggestive of adenomyosis that may be detected on ultrasound that can be categorized as direct or indirect as per the revised definitions of the Morphological Uterus Sonographic Assessment (MUSA).^60^ Direct features indicate the presence of ectopic endometrial tissue in the myometrium, including myometrial cysts, hypoechogenic islands, and echogenic subendometrial lines and buds. In contrast, indirect features are secondary to the ectopic endometrial tissue, in many cases reflecting the muscular hypertrophy and hyperplasia, and comprise a globular shape of the corpus, asymmetrical myometrial thickening, fan‐shaped shadowing, translesional vascularity, and an irregular or interrupted junctional zone.^60^

It is important to view adenomyosis on a spectrum, defined by the number and type of features, and not as a binary (present or absent) diagnosis. Different individuals may have different numbers and combinations of features. Still, differences in genetics, epigenetics, and molecular expressions within the ectopic endometrial tissue may contribute to the clinical picture seen in a given patient. For example, in work performed in the UK, a single feature of adenomyosis as determined by 2D TVUS was not associated with quantified HMB, but bleeding volume increased proportionally to the total number of features detected.^61^

Magnetic Resonance Imaging (MRI) is a more expensive technique to diagnose adenomyosis accurately and, in most instances, is used as a second‐line investigation with a sensitivity of 88%–93%.^62^ Whereas most MRI features of adenomyosis overlap those seen on TVUS, focusing on the junctional zone and irregularities within provides greater diagnostic potential for MRI.^62^, ^63^

#### Leiomyomas (AUB‐L; uterine fibroids)

Leiomyomas are prevalent benign tumors comprising smooth muscle cells and fibroblasts while being rich in extracellular matrix. Their prevalence increases with age and has been reported to be 69% in white women and 83% in black women by the age of 50.^64^ Whereas the exact pathogenesis of a leiomyoma is unknown, it appears to be multifactorial with the involvement of genetics, gonadal steroid exposure, and environmental factors.^65^, ^66^ Leiomyomas can manifest in various phenotypes that vary in size, number, and location. While most women have no symptoms related to their leiomyomas,^67^ others may present with a spectrum of symptoms. Consequently, decisions regarding expectant, medical, or procedural interventions depend on a combination of the desires of the individual and their leiomyoma phenotype. Collectively, these circumstances challenge investigators and clinicians to understand when and how a given leiomyoma phenotype is responsible for the experienced symptoms and what therapeutic options best fit the patient's goals, particularly those related to fertility.

**FIGURE 3:**
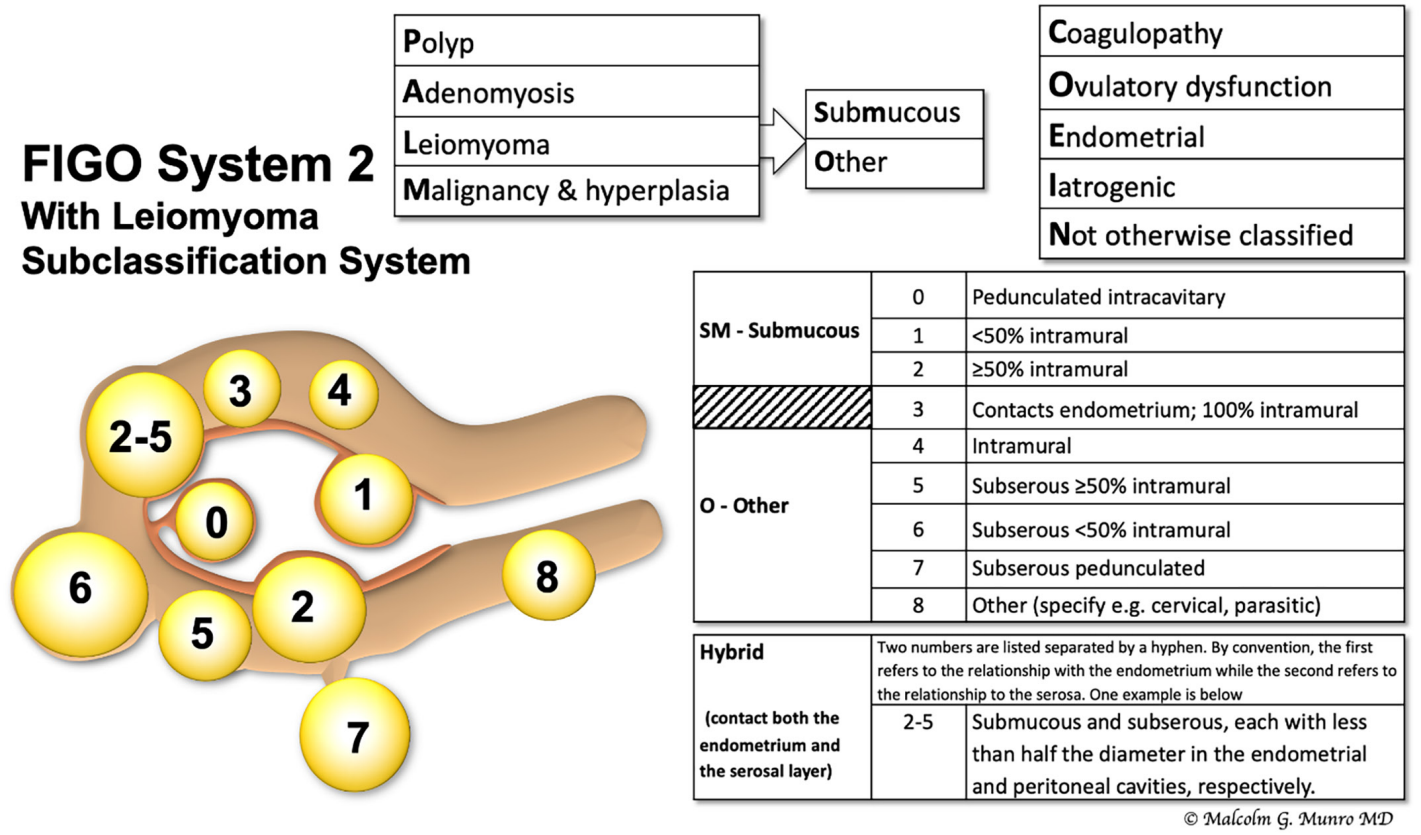
FIGO subclassification system for leiomyomas.^30^ The system includes the tertiary classification of leiomyomas dependent on location. Classification of lesions that are transmural are categorized by their relationship to both the endometrial and the serosal surfaces. The endometrial relationship is noted first, with the serosal relationship second (e.g. Type 2–5). Leiomyomas that do not relate to the myometrium at all are classified as Type 8, and would include cervical lesions (demonstrated), those that exist in the round or broad ligaments without direct attachment to the uterus, and other so‐called “parasitic” lesions.

The complex context created by this highly prevalent disorder led to the development of the FIGO classification system, which was designed primarily according to the location of the leiomyoma with respect to the endometrium and uterine serosa (Figure 3). Submucous leiomyomas include those in contact with the endometrium, as determined clinically by hysteroscopy or uterine imaging using ultrasound‐based techniques or MRI. While Types 0, 1, and 2 project into the endometrial cavity, Type 3 leiomyomas are those in contact with the endometrium without focal distortion of the endometrial cavity. Intramural leiomyomas are surrounded by myometrium and are deemed Type 4 leiomyomas. In contrast, those in contact with the uterine serosa (Types 5, 6, and 7) are categorized by the degree to which they project out of the uterus, with Type 7 being the pedunculated variety. Type 8 leiomyomas are those found in alternate locations, most commonly the cervix. Especially important for intrauterine surgery, such as hysteroscopic myomectomy, are the tumors that contact both the endometrium and the uterine serosa, which are categorized with two numbers reflecting, in order, the endometrial and serosal relationships (e.g. Types 2–5 or 3–6). It is critically important that clinicians do not attempt hysteroscopic myomectomy, as removal will result in peritoneal cavity entry, termination of the procedure, and the risk of damage to surrounding visceral and other structures including the bladder, bowel, ureters, and blood vessels.

Only submucous leiomyomas appear to be linked directly with objectively measured increased blood loss—data that have not distinguished if Type 3 lesions are included.^68^ Consequently, since most leiomyomas do not appear to contribute to AUB symptoms, women with Type 3–8 leiomyomas should be investigated for other possible causes of AUB. There is a paucity of data related to the leiomyoma‐related cause of AUB symptoms when there is no direct physical link with the endometrium.

TVUS remains the first line of investigation for leiomyomas, with a sensitivity of between 90% and 99%.^69^ Leiomyomas are typically well‐defined spheroids with shadows at the edge of the lesion caused by the interface between fibroid tissue and normal myometrium. The echogenicity of the lesion varies depending on the proportion of muscle cells and fibrous stroma within the lesion. Color Doppler can delineate circumferential flow around the lesion, sometimes characterized as a “ring of fire”.^70^ Sonohysterography can improve the accuracy of diagnosis when lesions are suspected to involve the endometrial cavity.^71^ Further information on the number, location, and size of the lesions may be provided by MRI and may suggest the possibility of sarcomatous change; however, diagnostic reliability for malignancy remains uncertain.^72^

#### Malignancy (AUB‐M)

When histopathologic examination of specimens taken from the uterine corpus of a reproductive‐aged woman with AUB reveals premalignant change or a frank malignancy, the woman is deemed to have AUB‐M. In most instances, specimens are an endometrial biopsy collected via a suction catheter (e.g. Pipelle CCD Mark II, Laboratoire CCD, France) or performed under hysteroscopic direction. Atypical endometrial hyperplasia, also known as endometrial intraepithelial neoplasia (EIN), is known to be a precursor for endometrial cancer and is included in this category.^73^, ^74^, ^75^ In general, FIGO follows the definitions and classifications of the World Health Organization (WHO) or the FIGO Oncology Committee. The WHO has recently updated The Cancer Genome Atlas for the classification of female genital tumors.^74^, ^76^

### 5.3 Relationship between pathogenesis and clinical investigations: Nonstructural causes of AUB

#### Coagulopathy (AUB‐C)

When AUB is determined to occur secondary to congenital or acquired systemic disorders of hemostasis, it is categorized as AUB‐C^30^, ^35^ with the most common disorder being von Willebrand disease.^25^ Coagulopathies such as von Willebrand disease exist on a spectrum, therefore mild cases detected biochemically may or may not contribute significantly to the symptoms experienced by the patient. Using a FIGO‐recommended screening tool (see Section 5) for coagulopathies, it is possible to understand if further investigation is needed, with or without the consultation of a hematologist or other clinician with expertise in coagulopathies.^35^, ^36^

#### Ovulatory dysfunction (AUB‐O)

**FIGURE 4:**
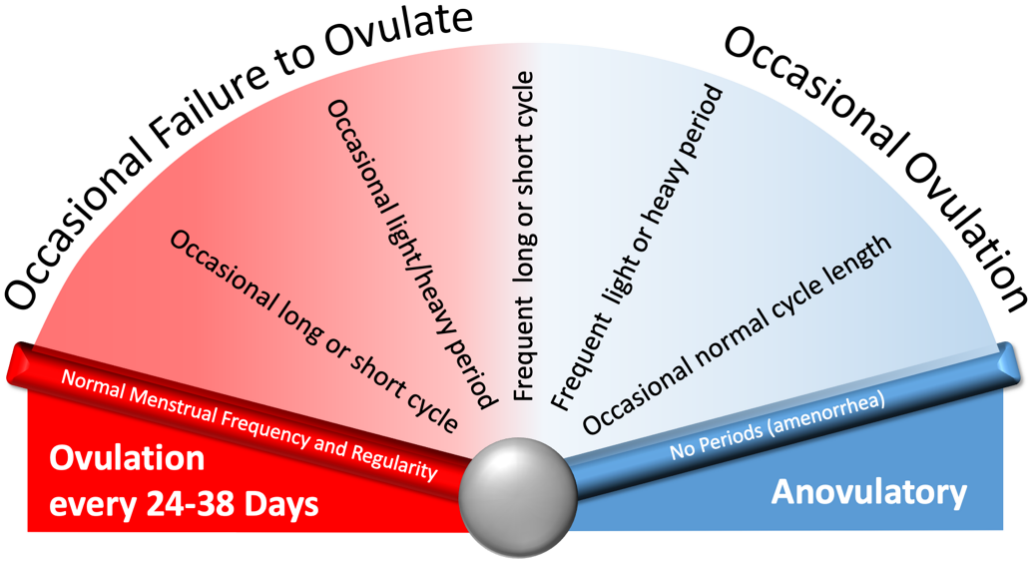
Typically, but not always, these disorders manifest abnormalities in FIGO System 1 parameters that describe menstrual bleeding pattern: frequency, regularity, duration and volume of bleeding.^79^, ^80^, ^81^

Ovulatory disorders exist on a spectrum (Figure 4) that encompasses occasional delayed or failed ovulation to a chronic process that can cause amenorrhea or manifest with bleeding that varies in volume, duration, and frequency.^77^, ^78^

Whereas the diagnosis of AUB‐O is generally suggested by a history of irregular bleeding obtained via FIGO's AUB System 1, the specific cause of the ovulatory disorder requires a structured evaluation. While ovulatory status can usually be suggested from FIGO System 1, when there is a lack of clarity other measures may be employed, including daily measurement of basal body temperature or obtaining serum progesterone in the suspected luteal phase of the cycle.^35^

**FIGURE 5:**
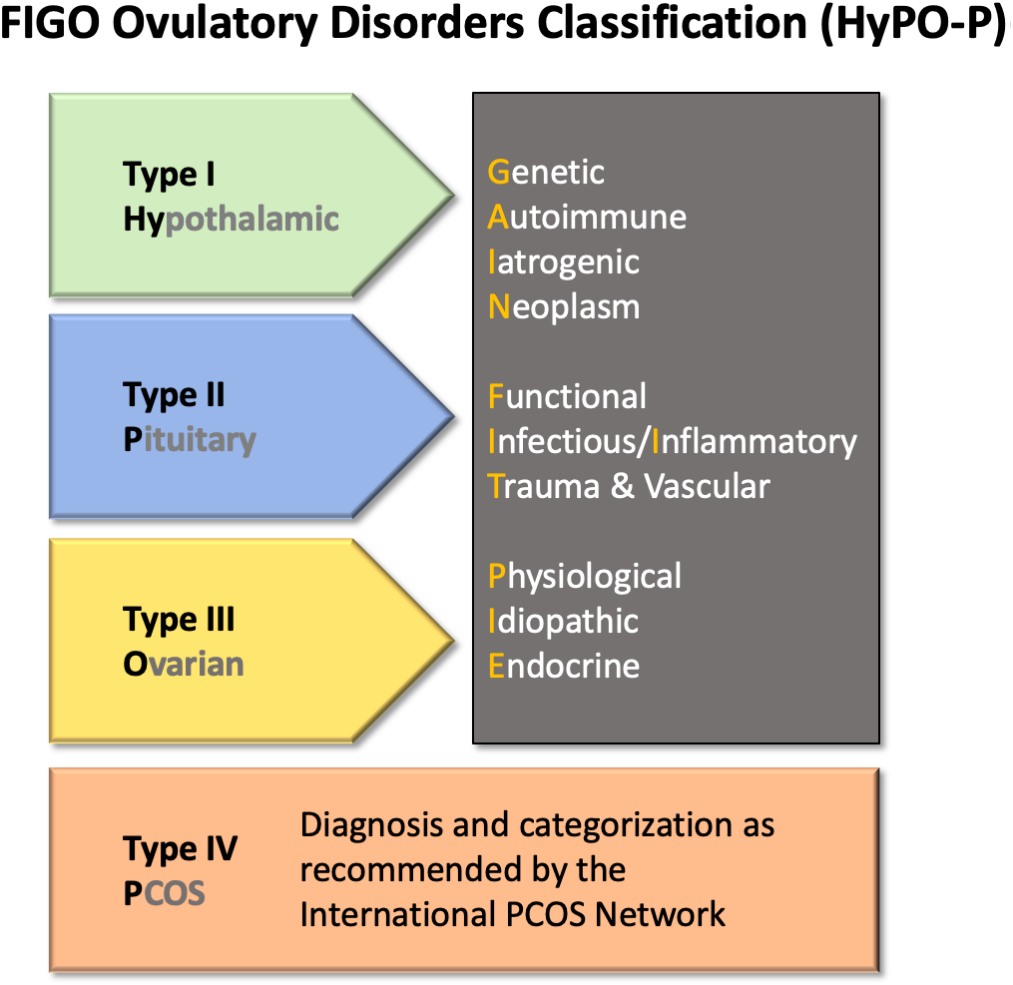
The proposed FIGO classification system for ovulatory disorders.^79^, ^80^, ^81^ This system allows for an initial allocation of presumed primary source of ovulatory dysfunction (i.e. hypothalamus, pituitary gland, or ovary). Polycystic ovary syndrome (PCOS) is defined separately and the WHO classification system for the same should be adopted. After anatomical allocation, known or presumed mechanism is classified according to the GAIN‐FIT‐PIE acronym, as appropriate and applicable.

Recently, a classification system for ovulatory disorders was developed under the aegis of FIGO by the Committees for Menstrual Disorders and Related Health Impacts (formerly the Menstrual Disorders Committee) and Reproductive Endocrinology and Infertility.^79^, ^80^, ^81^ The development involved journal editors, recognized experts, and national, subspecialty, and lay societies from around the world to achieve international consensus for replacing a system developed over 50 years ago by the WHO. The new system comprises three anatomic categories, as well as a separate category for polycystic ovary syndrome (PCOS), which are recognized in the acronym “HyPO‐P”: Type 1 (Hypothalamic); Type 2 (Pituitary); Type 3 (Ovarian); and Type 4 (Polycystic Ovary Syndrome) (Figure 5). Each of the three anatomic categories includes a list of subtypes that follow the acronym GAIN‐FIT‐PIE (Genetic, Autoimmune, Iatrogenic, Neoplasm, Functional, Infectious and Inflammatory, Trauma, and Vascular; Physiological, Idiopathic, Endocrine) reflecting the various possible mechanisms within a given anatomical location. The investigational pathway for an individual with an ovulatory disorder is dependent upon a number of factors, including age, reproductive goals, and the suspected causes based on the initial evaluation.

#### Endometrial disorders (AUB‐E)

The category of primary endometrial disorders is assigned to presumed ovulatory women with AUB for whom there is no other demonstrable cause. While likely extremely common, the diagnosis of AUB‐E may be difficult to make for a number of reasons. The most obvious case is the symptom of HMB in the context of an entirely normal investigation. However, AUB‐E may occur when the evaluation demonstrates asymptomatic abnormalities such as a leiomyoma not in contact with the endometrium, or a single adenomyosis feature, neither of which is likely to cause the symptom of heavy bleeding.

Fundamentally, these disorders of endometrial hemostasis reflect primary dysfunction in the mechanisms by which menstrual bleeding is controlled locally that likely comprise some combination of deficiencies in the mechanisms of vasoconstriction, alterations in the formation or maintenance of intravascular thrombi, and impairment in the process of endometrial repair.^3^, ^12^ Unfortunately, while there exists almost half a century of study of these molecular mechanisms in women with (what we are calling) AUB‐E, there are currently no diagnostic tests available to clinicians, making it necessary to make a presumed diagnosis of this category. This circumstance may contribute to what is likely a vast underdiagnosis and normalization of the symptoms of women with AUB‐E.

#### Iatrogenic (AUB‐I)

There are many mechanisms through which medications or other interventions may lead to AUB. These include medicated or inert intrauterine devices, drugs that interfere with the coagulation cascade, as well as medication that interferes with ovulatory mechanisms.^30^, ^35^ Women using gonadal steroids for contraception or the treatment of AUB often experience breakthrough bleeding or unscheduled bleeding that should be captured in FIGO System 1. When AUB is suspected to be caused by a device or medication, it is crucial to ensure that the bleeding originates from the endometrial cavity, and then further investigate, manage, and counsel the patient appropriately. Clinical guidance on the management of abnormal bleeding while using gonadal steroids has been reviewed elsewhere^82^, ^83^; however, it is essential to remember that failed medical management of AUB (including failed benefit of gonadal steroids) is a risk factor for malignancy and should be investigated, ideally with endometrial sampling and histopathologic analysis.^84^

#### Not otherwise classified (AUB‐N)

There exist other entities within or affecting the uterus that may lead to AUB; however, many are rare, have an unclear relationship, or are otherwise not included in FIGO System 2. Therefore, these possible causes or contributors to AUB symptoms have been categorized as not otherwise classified or AUB‐N. It is quite possible that one or more of these diagnoses may be reassigned to a new or existing category in the future.

At present, AUB‐N includes arteriovenous malformations and cesarean scar defects. Uterine arteriovenous malformations are abnormal connections between uterine arteries and veins. They are rare, can be congenital or acquired, and usually occur in women of reproductive age or after pregnancy. They represent a potential cause of life‐threatening bleeding. Diagnostic imaging can include ultrasound, computerized tomography (CT), MRI, and digital subtraction angiography; however, the appropriate technique depends on the patient's clinical situation and comorbidities. As an acute cause of AUB, clinical management focuses primarily on gaining hemodynamic stability, after which definitive management depends on the age of the patient and fertility desires.^85^

The high prevalence of cesarean scar defects reflects the high and increasing prevalence of cesarean section, which the WHO estimates currently at 20%, with an expected increase to one in three by 2030.^86^ The cesarean scar defect tends to be located in the anterior wall of the uterus, at the site of the hysterotomy incision, and has the potential to hold menstrual blood within the defect leading to a unique pattern of early‐cycle intermenstrual bleeding and prolonged menstruation recently described in a systematic review and meta‐analysis.^34^ FIGO System 1 can be used to identify this bleeding pattern and the diagnosis can be made by one or a combination of TVUS, sonohysterography, MRI, and hysteroscopy.

## 6 DIAGNOSTIC STEPS FOR AUB WITH/WITHOUT ID/IDA

Before assuming that women have acute or chronic nongestational AUB, it is essential to assess for pregnancy with appropriate testing and examine the exocervix, vagina, vulva, perineum, perianal, and other potentially confounding locations. While we have not focused on acute AUB in this paper, the presence of urgent or emergent very heavy bleeding should lead to standard approaches for hemodynamic instability as well as the use of techniques and pharmaceutical agents designed to stop the loss of blood.^87^

For those with chronic AUB, the clinician should take the time to obtain the detailed history required for FIGO System 1 and to evaluate appropriately to determine what category or categories of FIGO System 2 apply to the patient. System 1 is a necessary gateway to System 2. It is important to consider all the PALM‐COEIN causes for each patient, as there may be more than one contributing factor. In such cases, an individual may be documented as having, for example, AUB‐L, ‐A, ‐O if the investigation demonstrates leiomyomas, evidence of adenomyosis, and a bleeding pattern suggestive of an ovulatory disorder.

The use of blood tests, imaging investigations, endometrial sampling, and hysteroscopy should be considered in the context of the specific patient. For example, hysteroscopy, endometrial sampling, and uterine imaging are not necessary to manage an otherwise normal 19‐year‐old with presumed AUB‐O. Still, this patient might benefit from laboratory evaluation of their thyroid, iron, and anemia status and discussing their situation concerning psychological stress and exercise. On the other hand, a 49‐year‐old with a similar history may require uterine imaging and endometrial sampling. A tailored adaptive approach may be essential but common to all with the symptom of HMB. Assessment of iron status is discussed elsewhere in this Supplement.^88^

## 7 EVIDENCE GAPS

### 7.1 Abnormal uterine bleeding and female technology (FemTech)

In recent years, there has been an overall development of female technology designed to assist with women's health. Examples include software programs to track fertility or menstrual cycles and hardware devices such as skin‐worn sensors and intelligent technology menstrual cups. Frameworks are being developed to encourage these products to be inclusive.^89^ These products can potentially improve the characterization and diagnosis of causes of AUB; for example, ovulatory disorders and contributing factors.^90^ Recent advances in estimation of menstrual blood loss via digital applications may also help women understand their symptoms, encourage presentation to healthcare professionals, and help elucidate the true prevalence of AUB in the reproductive years.^91^

### 7.2 Role of iron in menstrual physiology

The need to monitor AUB patients for ID or IDA is an important clinical consideration. It has been found that iron plays a crucial role in the repair of injured mucosal surfaces^92^; however, physiologically, iron's role in endometrial repair and thus the menstrual cycle is yet to be determined.

### 7.3 AUB and COVID‐19

During the recent COVID‐19 pandemic, clinicians were required to rapidly adapt their modes of delivery of clinical care, including the evaluation of women with AUB.^93^ As the world returns to normal clinical activities, clinicians must not lose sight of how this remote care has impacted women overall and the need to ensure thorough clinical assessment on returning to face‐to‐face appointments. Evidence is also accruing concerning the impact of the SARS‐Cov‐2 infection and related vaccines on the menstrual cycle. It has been reported that women infected with SARS‐Cov‐2 experienced a decrease in menstrual volume, and although the majority of women studied had an unchanged menstrual cycle length, 19% experienced a prolonged cycle^94^; however, the authors do not state the exact number of days of prolongation of the cycle, making it difficult to understand the impact on FIGO System 1 parameters. These changes were reported to be transient in most patients.^95^ After the COVID‐19 vaccine, menstrual cycle changes have been reported; for example, an increase in cycle length by one day^96^ compared with an unvaccinated cohort. However, more research is needed.^97^

In the UK, via the yellow card reporting system for adverse events related to medication,^98^ there have been reports of HMB, increased frequency of bleeding, and IMB after the COVID vaccine. However, the ability to report menstrual disturbances is confusing and currently uses terminology that is obsolete and not accessible; for example, hypomenorrhea or oligomenorrhea. Of note, those reporting can also choose to report “Abnormal Uterine Bleeding,” which would include many other bleeding patterns listed on the reporting site. Adopting FIGO System 1 is crucial in ensuring reporting and classification of menstrual irregularities. The mechanisms behind the menstrual cycle changes reported in relation to COVID‐19 infection or the COVID‐19 vaccine are yet to be elucidated, as well as the length of time it takes for menstrual bleeding patterns to return to normal. Where this infection and its vaccines fit into the AUB FIGO classification system is yet to be determined.

## 8 FINAL THOUGHTS

Abnormal uterine bleeding and its associated conditions (e.g. ID and IDA) are underrecognized and underreported. By utilizing FIGO System 1, information about menstrual cycles and bleeding patterns can be gathered in a concise and straightforward manner. This provides the gateway to a complete evaluation with appropriate investigations and allows for a diagnosis comprising one or more elements in FIGO System 2. Menstruation and menstrual cycle symptoms can provide valuable insights into the overall health of women and girls.^15^ To date, there has been insufficient attention to inquiry about menstruation and menstrual cycle symptoms. Therefore, a conscious effort to include questions surrounding menstrual health, mirroring FIGO Systems 1 and 2, as well as signs and symptoms of associated ID/IDA, in all clinical trials, including women and girls of reproductive age, could significantly improve detection and onward management of these debilitating conditions.

## AUTHOR CONTRIBUTIONS

All authors were involved in the design and write‐up of this article.

## CONFLICT OF INTEREST STATEMENT

HODC has received clinical research support for laboratory consumables and staff from Bayer AG (paid to institution) and provides consultancy advice (paid to institution) to Bayer AG, PregLem SA, Gedeon Richter, Vifor Pharma UK Ltd, AbbVie Inc., and Myovant Sciences GmbH. HODC has received royalties from UpToDate for an article on abnormal uterine bleeding. VJ receives salary and research consumables support from Wellbeing of Women (WoW). MGM reports a consultancy role with the following entities: Abbvie Inc, American Regent Inc, Daiichi Sankyo Ltd, Hologic Inc, Myovant Sciences, Pharmacosmos A/S, Vifor, and indirect research funding from Abbvie Inc and Pharmacosmos.

## Data Availability

Data sharing is not applicable to this article as no new data were created or analyzed in this study.
